# Dynamic, adaptive sampling during nanopore sequencing using Bayesian experimental design

**DOI:** 10.1038/s41587-022-01580-z

**Published:** 2023-01-02

**Authors:** Lukas Weilguny, Nicola De Maio, Rory Munro, Charlotte Manser, Ewan Birney, Matthew Loose, Nick Goldman

**Affiliations:** 1 European Molecular Biology Laboratory, European Bioinformatics Institute, Wellcome Genome Campus, Hinxton, UK; 2 DeepSeq, School of Life Sciences, Queen’s Medical Centre, University of Nottingham, Nottingham, UK; 3 Present Address: Department of Life Sciences, Imperial College London, London, UK

**Keywords:** Data acquisition, Genetics research, Next-generation sequencing, Next-generation sequencing, Next-generation sequencing

## Abstract

Nanopore sequencers can select which DNA molecules to sequence, rejecting a molecule after analysis of a small initial part. Currently, selection is based on predetermined regions of interest that remain constant throughout an experiment. Sequencing efforts, thus, cannot be re-focused on molecules likely contributing most to experimental success. Here we present BOSS-RUNS, an algorithmic framework and software to generate dynamically updated decision strategies. We quantify uncertainty at each genome position with real-time updates from data already observed. For each DNA fragment, we decide whether the expected decrease in uncertainty that it would provide warrants fully sequencing it, thus optimizing information gain. BOSS-RUNS mitigates coverage bias between and within members of a microbial community, leading to improved variant calling; for example, low-coverage sites of a species at 1% abundance were reduced by 87.5%, with 12.5% more single-nucleotide polymorphisms detected. Such data-driven updates to molecule selection are applicable to many sequencing scenarios, such as enriching for regions with increased divergence or low coverage, reducing time-to-answer.

## Abstract (web summary)

Nanopore selective sequencing with real-time decision updates mitigates coverage bias.

## Main

Long-read sequencing provides the ability to generate reliable reads consisting of multiple kilobases or even megabases^1^. Such ultra-long reads are highly useful for many genomics applications—for example, increasing assembly contiguity, even allowing the construction of telomere-to-telomere assemblies^2,3^; interrogating variation in hard-to-decipher regions of a genome, such as repeats, centromeres or segmental duplications^4^; or generating chromosome-level epigenetic maps^5^.

One way of generating long reads is through the use of nanopores. This concept, first explored in the 1980s, was commercialized by Oxford Nanopore Technologies (ONT)^6^. It relies on the idea of using a protein nanopore as a biosensor, permitting measurement of fluctuations of an ionic current across the pore caused by the presence of nucleotides of a translocating DNA or RNA molecule. Single-molecule sequencing is possible without the need for prior amplification and can also be used to directly read RNA without reverse transcription^7^. The generation of sequencing reads in real time, which, in combination with fast library preparation, immensely reduces the time needed to go from biological sample to data analysis, enables (for example) intraoperative decision-making^8^, improved global food security by rapid identification of plant viruses^9^ and portable genomic surveillance^10^. Over the past years, nanopore sequencing error rates have decreased to ~1%^11^, approaching the accuracy of short-read platforms.

A unique feature of nanopore sequencing is the possibility to reverse the voltage across the pores to reject fragments before reading them in their entirety, termed adaptive sampling or ‘Read Until’^12,13^. This enables selection of molecules for sequencing based on real-time assessment of a small initial part of a read rather than complex sample preparation. Initially, identifying fragments’ genomic origin was achieved by matching the electrical signal directly to reference genomes translated into simulated current traces. Recent improvements, however, harness the computing power of GPUs for real-time basecalling, making it possible to use optimized bioinformatics tools for further processing—for example, read mapping^14^. This has led to much interest in experiments that can be aided by real-time selection of molecules for sequencing (see, for example, refs. ^15–18^).

In current implementations, decisions about which fragments to read or reject are based on a priori decisions—for example, of regions of interest (ROIs) in a genome^12,14^. This restricts their application to a narrow range of problems where sufficient information is available in advance of sequencing a potentially poorly characterized sample. We hypothesized that such decisions could also incorporate information obtained from already sequenced fragments generated in the current sequencing run.

**Fig. 1:**
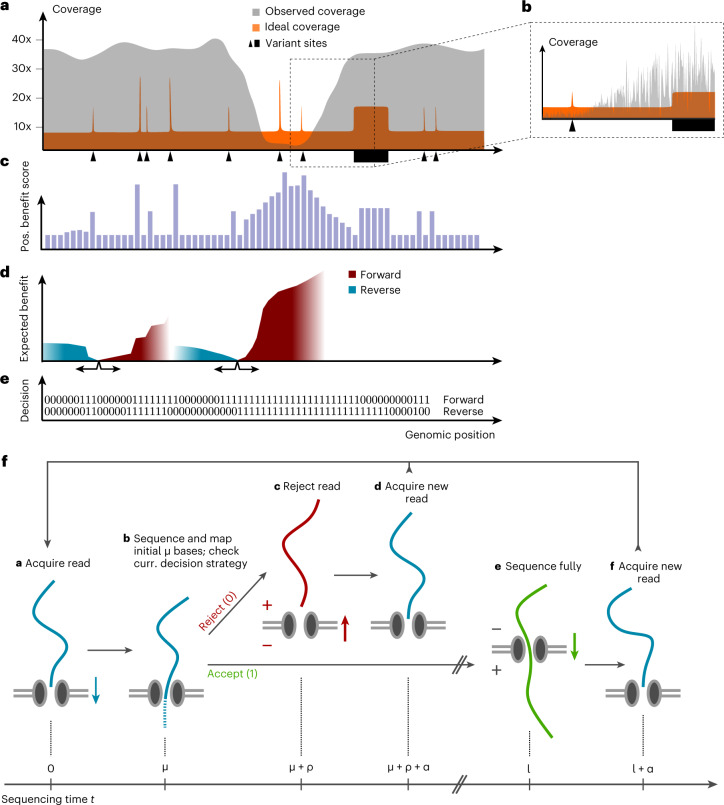
Methodological overview of dynamic, active sampling. **a**, Different sites might require different levels of coverage; for example, sites lacking variation are resolved by few reads, and sites of particular interest require more. Accumulation of coverage beyond that necessary (observed coverage in gray, exceeding ideal coverage in orange) is wasteful, whereas other sites would benefit from observing more data (observed < ideal). **b**, Local fluctuations in the distribution of fragment origins also result in uneven coverage and reduced efficiency of sequencing. **c**, We quantify the genotype uncertainty at each site based on prior probabilities and data observed so far. The expected shift in uncertainty caused by observing a new read at that position is expressed as ‘positional benefit score’. **d**, The expected benefit of a hypothetical read starting at each location is computed as the sum of accumulated positional scores, weighted by the probability of reaching those positions, illustrated for forward and reverse reads starting at two positions. **e**, A Boolean decision strategy for each position instructs the sequencer to either continue sequencing (1) or reject from the pore (0) a read that starts at that position. Stages **c**–**e** are updated and iterated throughout the sequencing experiment. **f**, Overview of our model of the sequencing process. A novel read is acquired, and, after sequencing its initial bases, its starting position and orientation are identified, determining its fate according to the current decision strategy (**e**). Upon rejection (upper path), the pore is freed, a new read is acquired and the model iterates from the beginning. Conversely, upon acceptance (lower path), the molecule translocates through the pore until all of its nucleotides are read. New read acquisition and model iteration then proceed as before.

During a sequencing experiment, the distribution of coverage depth might not correspond well to the requirements of the experiment—for example, when determining variant sites (Fig. 1). Commonly, at present, the overall coverage would have to be increased to ensure sufficient sampling throughout, causing wasteful data acquisition in regions that are not of continued interest. We address this issue by generating dynamic decision strategies that redistribute coverage to positions of greatest value at any time during an experiment. Our method can focus sequencing on variant sites, without a priori knowledge of their location, increasing the accuracy of called genotypes. Furthermore, it can divert sequencing resources away from regions with high coverage toward regions with low coverage, leading to more homogeneous distribution of sequencing reads.

To summarize, our approach of dynamic, adaptive sampling allows us to change what is sampled during sequencing in light of the already observed data, maximizing the information gain and ultimately leading to various potential advantages, such as reduced time-to-answer and increased confidence in called genotypes. We demonstrate our method by mitigating coverage bias in a microbial mock community, leading to higher coverage depth of low-abundance species, an increased limit of detection and improved variant calling.

## Results

### Model and implementation

#### Probability distributions of genotypes quantify uncertainty

We present a method that enables dynamic decision strategies during sequencing using nanopores. By calling it ‘dynamic’, we emphasize our extension of current approaches, which are limited to a priori choice of target regions. In this section, we give an overview of the methodology, with further details and formal explanations provided in the Methods and Supplementary Methods.

First, we capture the amount of information at each site of one or multiple genomes by considering a probability distribution over all possible genotypes. The prior of this distribution can be informed by reference genomes (in the sense of any assembly) and is subsequently updated as we collect data throughout the experiment—that is, we calculate a posterior probability distribution based on the observed nucleotides at that position. Additionally, ploidy and sequencing error probabilities are taken into account. This allows us to calculate the remaining uncertainty about the genotype at each site and how much information we might gain from one further read covering that site, which we call the ‘positional benefit score’ (Fig. 1c). Broadly speaking, positions that are already covered by many agreeing reads will receive a low score; conversely, positions covered by few, or contradictory, reads will score highly, as individual observations have higher potential to influence the posterior distribution.

#### Quantifying the information content of sequencing reads

Reads are derived from contiguous sections of a genome, so we combine these scores over adjacent sites to estimate the expected information gain from a sequencing read. This is based on starting location and orientation as well as the distribution of previously observed read lengths (Fig. 1d). Ultimately, a sequencing read that is expected to give a higher sum of scores—that is, a greater reduction in the uncertainty of genotypes at the positions it covers—will be considered more useful than a read with limited potential to alter the site-wise posterior probabilities.

Using the expected benefit of reads, we can define criteria for making decisions about which fragments to sequence fully and which to reject from nanopores. Note that, in line with common usage, we refer to DNA molecules and their translation into sequence space interchangeably as ‘fragments’ and ‘reads’. Our aim is to optimize the rate of accumulation of information—that is, of expected benefit—across all pores and over time. As we collect data throughout the sequencing experiment, the value of reads at different positions will change, and, therefore, the decision strategy adapts to these changes dynamically in real time. The strategies are found by ranking sites according to their expected read benefit, taking into account the expected time of sequencing them (Fig. 1f). This way, we can calculate the optimal subset of sites to accept reads from, to increase the gain of benefit at that moment in the experiment. Resulting strategies are stored as Boolean vectors, indicating the intended decision about a read starting at any genomic position (Fig. 1e).

We call our approach of finding an optimal strategy BOSS-RUNS: ‘Benefit-Optimising Short-term Strategy for Read Until Nanopore Sequencing’. Further methodological details, overview of parameters and variables in the model and proof of optimality are given in the Methods, in Supplementary Methods and in Supplementary Table 1.

#### Real-time implementation

BOSS-RUNS is implemented in Python, available at https://github.com/goldman-gp-ebi/BOSS-RUNS, and interacts with the sequencing device through readfish^14^ and the Read Until API^13^. BOSS-RUNS periodically includes all new data by mapping newly observed basecalled reads to one or more reference genomes using minimap2 (ref. ^19^).

### Dynamic enrichment of differentially abundant species

#### Experimental setup

Enrichment of ROIs by rejecting unwanted reads was previously demonstrated^12,14,20^. BOSS-RUNS can be applied more generally and makes use of targeted rejections even in the absence of specific ROIs. Here, we consider a scenario of whole-genome resequencing where the entire genome is considered of interest, and we showcase a scenario with ROIs in Supplementary Results, Section 2.

**Fig. 2:**
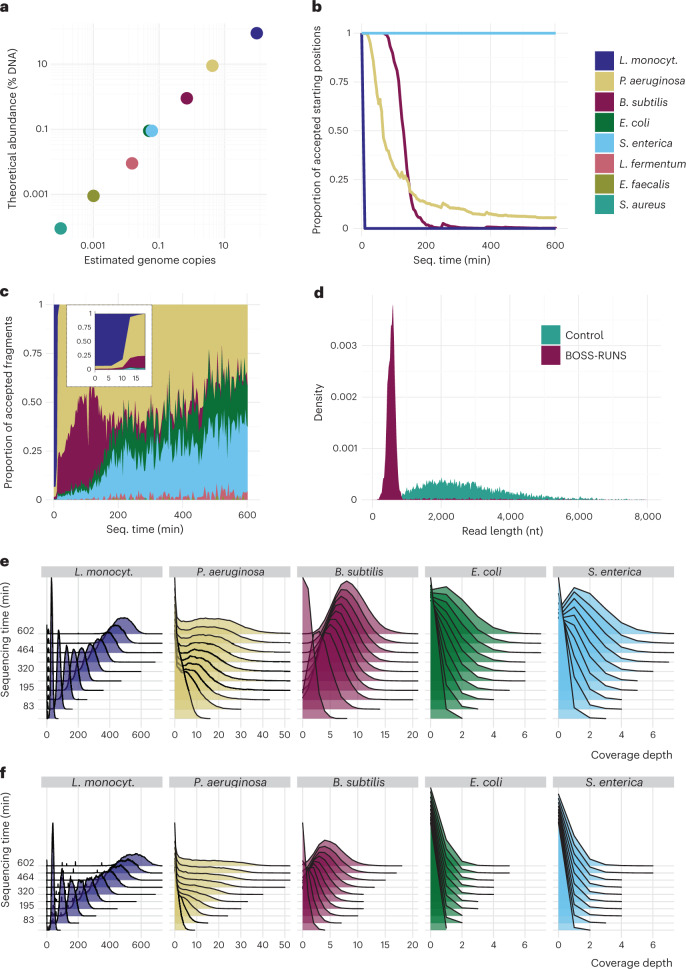
BOSS-RUNS strategy adapts during sequencing of the Zymo bacterial mixture. **a**, We sequenced eight bacterial species of the ZymoBIOMICS mixture with logarithmically distributed abundances covering seven orders of magnitude. Colors correspond to species as in **b**,**c** and **e**,**f**. **b**, After initially accepting any read from any considered genome, we quickly observe rejections from the most abundant bacteria, *L. monocytogenes*, followed by *P. aeruginosa* and *B. subtilis*. The plot shows the proportion of accepted positions in each speciesʼ genome over the duration of the experiment. **c**, The proportion of accepted fragments that derive from each bacterial strain demonstrates the effect of the changing decision strategy. The inset plot shows how the strategy rejects almost all *L. monocytogenes* reads after the first 10 minutes. **d**, The distribution of read lengths confirms that BOSS-RUNS rejects most sequencing reads, with a clear peak corresponding to rejected reads. Coverage distribution using BOSS-RUNS (**e**) shows depletion of DNA from more abundant genomes in turn for enrichment of rare species when compared to the control section on the flowcell (**f**). Accumulation of coverage over time is shown by the distributionsʼ shift to the right within panels. Results from the three least abundant species are omitted owing to non-obvious differences in this type of visualization.

One situation where the possibility of redistributing data is very effective is in the presence of coverage bias, either within or across genomes. Our first experiment has two major goals: to mitigate coverage bias across multiple differentially abundant genomes and to demonstrate that our ‘dynamic’ approach can increase sampling from variant or difficult-to-resolve sites without prior knowledge of their location. Therefore, we sequenced eight bacterial species of the ZymoBIOMICS microbial mixture (ZymoBIOMICS DNA Standard II D6311, Zymo Research) with logarithmically distributed abundances (the most abundant species comprising 90% of total DNA, the second most abundant species comprising approximately 9%, the third most abundant species comprising 1%, etc.; Fig. 2a). To measure the performance of BOSS-RUNS against a control sequencing run, we divided the available pores on a single flowcell into two sets and ran BOSS-RUNS on one set, whereas we performed no rejections on the other.

To mimic a realistic sequencing experiment where the exact bacterial strains are unknown, we used reference assemblies of closely related strains (Methods). This also allowed us to evaluate how our method focuses on sites that differ between reference and experimental sample.

#### BOSS-RUNS strategy

During sequencing, we can observe how the decision strategy changes over time. As the genomes of individual bacteria are continuously resolved—that is, we become more certain about the genotype at many sites—the proportion of positions at which we still require more information decreases. Due to the differential abundance of the sample species, *Listeria monocytogenes* is considered mostly resolved after only a few minutes, followed later by *Pseudomonas aeruginosa* and *Bacillus subtilis* (Fig. 2b). Accordingly, the proportion of accepted reads demonstrates that the focus switches from the most abundant bacteria toward rarer species (Fig. 2c). As in ref. ^14^, all species’ abundances can still be accurately quantified by considering the total number of observed reads per species (Supplementary Fig. 1).

The rate at which individual genomes are resolved is not equal across all bacteria. For example, the proportion of accepted sites in *L. monocytogenes* or *B. subtilis* decreases to values close to 0, whereas *P. aeruginosa* approaches a level of ~5.8% and does so at a slower rate. In other words, some sites of *P. aeruginosa* require more data to be confidently resolved, and a portion of sites remains uncertain despite sampling data throughout the run. This is due, in part, to different levels of large-scale variants—that is, insertions and deletions—between the strains in the Zymo community and the reference genomes we used and, in part, to differential coverage bias within each species’ genome.

Given the large difference in abundance and the prompt resolution of *L. monocytogenes*, we expect most sequencing reads to be rejected throughout the experiment. Indeed, BOSS-RUNS ejects most molecules after initial assessment, resulting in a peak of observed read lengths at ~480 bp (Fig. 2d). When splitting the sequencing data by target species, we observe a separation of the read length distribution into rejected and full-length reads that corresponds to expectations given the proportion of rejected reads from each species (Supplementary Fig. 2). The presence of a similar peak, even for rare species, indicates that some reads are also rejected. Most of these false rejections (84%) were due to inability to determine the source species from the initial fragment.

#### Improved sequencing of bacterial species

The effect of the changing decision strategy becomes evident when looking at the distribution of coverage depth over time. Coverage from the most abundant species is effectively redistributed to the scarcer species compared to the control (Fig. 2e,f). For example, for *Escherichia coli* and *Salmonella enterica*, which comprise only 0.1% of the input DNA, we achieve 3.9 and 4.0 times higher total yield compared to the control.

**Fig. 3:**
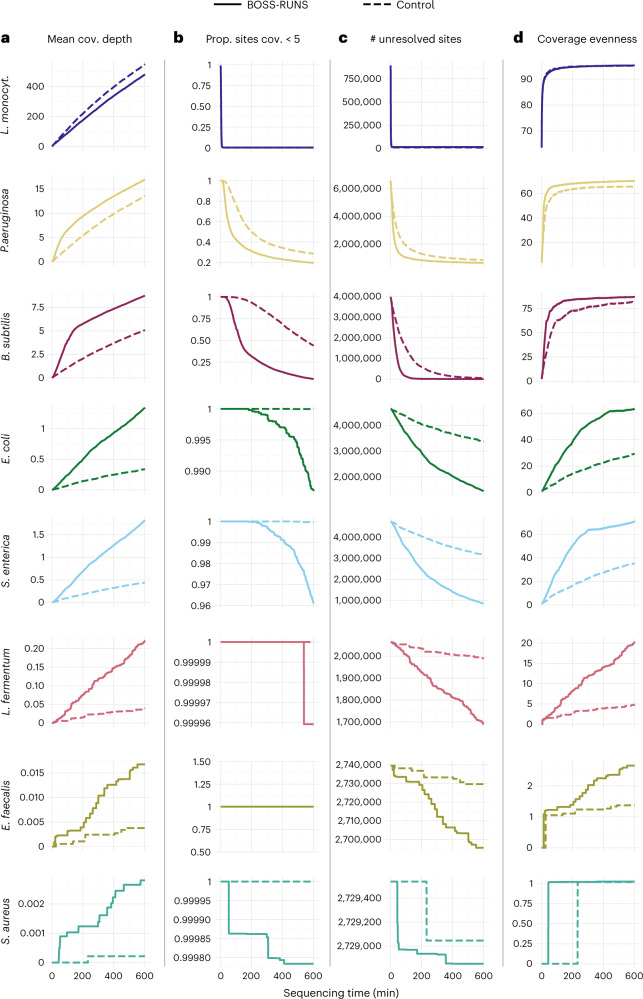
Improvements in sequencing a bacterial community using BOSS-RUNS. Four statistics highlight advantages of BOSS-RUNS (solid lines) compared to control (dashed lines) in an experiment lasting 600 minutes. **a**, Mean coverage depth over time. Coverage of the most abundant species is traded-off to collect more data from rarer species. As other genomes become resolved, a change in the rate of data accumulation is visible—for example, after ~180 minutes for *B. subtilis*. **b**, Reductions in the proportion of sites at <5× reveals that data are redistributed to areas of low coverage. **c**, Classifying sites as resolved if the posterior probability of one genotype is >0.99, we see that BOSS-RUNS achieves fewer unresolved sites owing to both sampling more data from rarer species and redistributing data within each genome. **d**, By focusing sequencing on sites with low coverage, BOSS-RUNS gives more even distributions of coverage. Note the different scales on the *y* axes to allow for sampling statistics of species of widely varying abundances.

Changes in mean coverage over time confirm these observations. Sacrificing data from heavily sampled organisms enables us to obtain more DNA from rare species (Fig. 3a). For example, BOSS-RUNS achieves between 4.1 and 5.8 times higher average coverage of the scarce bacteria. The proportion of low-coverage sites (<5×) also highlights the advantage of our method. This quantity decreases quicker, and reaches lower final levels, compared to the control for all but the most abundant genome (Fig. 3b). The redistribution of data from regions already well covered to areas of low coverage is one of the main features of BOSS-RUNS. In the case of *B. subtilis*, for example, this leads to less than 5% of sites with coverage less than 5× with BOSS-RUNS, against ~44% for the control. In rare species, this improvement in sites at coverage >5× was not caused by reads mapping to repeats or other low-complexity regions (Supplementary Table 2).

Classifying individual sites as resolved when the posterior probability of one genotype at a site surpasses 0.99, we can count the sites that still require more data to reach that level of certainty. Again, BOSS-RUNS shows better performance by reaching lower numbers of unresolved sites in a shorter time (Fig. 3c).

**Fig. 4:**
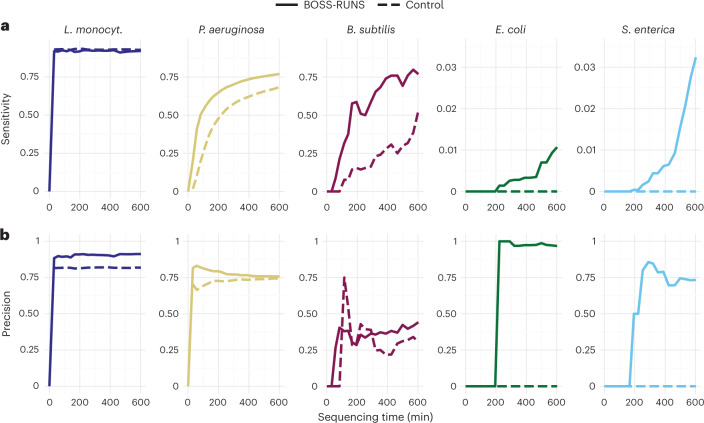
Dynamic, adaptive sampling leads to improved SNP discovery. We compared the variants called from data collected by the control (dashed lines) and by BOSS-RUNS (solid lines) to ground truth variants from deep, short-read sequencing of the same strains. Performing variant discovery at different timepoints gives further insight into the advantages of our method. **a**, Whereas the sensitivity of BOSS-RUNS is slightly lower for the most abundant species, we observe a larger number of discovered true positives in all remaining genomes. To highlight differences, we set the *y*-axis ranges to 0–0.95 for the first three species and 0–0.035 for the remaining two. **b**, The precision of variants called from data generated using BOSS-RUNS is at a similar level to the control or moderately higher.

Balancing coverage bias across genomes is not the only benefit: data are also redistributed within individual genomes. This effect is partly responsible for the gains described so far but may be somewhat concealed by species abundance differences. Using a measure of evenness that describes the uniformity of coverage distribution and is relatively independent of the absolute coverage^21^, we observe that BOSS-RUNS not only boosts the coverage of rare species but also ensures that coverage is more uniform within species, including those of higher abundance (Fig. 3d; for example, *P. aeruginosa* and *B. subtilis*). Even in cases where the total collected coverage of a strain is lower, it is possible that more uniform distribution of coverage could achieve a more desirable outcome of the experiment. Although in our experiment this effect is not readily visible in Fig. 3 for *L. monocytogenes*, we note that the improved precision of single-nucleotide polymorphism (SNP) detection for this species (see below; Fig. 4b) could be due to these effects.

#### Redistributing coverage to undersampled sites

Another way to explore the redistribution of data within genomes is to examine the already observed coverage at the sites that a read maps to when the decision about that read was made. Because our method focuses on reads from areas of highest uncertainty, we expect the mean and minimum coverage at sites spanned by accepted reads to be lower than at sites spanned by rejected reads. Indeed, these expectations were confirmed, emphasizing that BOSS-RUNS focuses on reads not only due to the abundance difference but also due to coverage variation within genomes and continues to sample from uncertain areas even after most of a species’ genome has been resolved (Supplementary Fig. 3)

#### Focused sequencing leads to improved variant calls

Next, we sought to perform variant calling for five of the bacterial species. (We excluded the three least abundant species, as we did not collect enough data to make reliable calls.) With this analysis, we tried to answer (1) whether we could successfully sample data from rare species to better identify variants and (2) whether BOSS-RUNS can effectively focus on sites where we observe variation and, therefore, increased uncertainty.

Our analysis is based on comparing inferred variants from data accumulated using BOSS-RUNS (or the control) to a ground truth derived from deep, short-read sequencing of the same strains (Methods). By making comparisons at multiple timepoints, we show how knowledge of variants accumulates over time (Fig. 4), which, in the future, could be used to optimize the duration of experiments needed to achieve particular levels of accuracy. For the most abundant species, *L. monocytogenes*, the decreased coverage with BOSS-RUNS leads to marginally lower sensitivity than for the control case. Nevertheless, high sensitivity is achieved in a very short time, and the effective redistribution of coverage within this species’ genome leads to increased precision. In turn, however, for all other species, the increased and better-targeted coverage means that more variants are discovered, with improved sensitivity and precision compared to the control sequencing without read rejections.

Even for the two bacteria, *P. aeruginosa* and *B. subtilis*, which are considered mostly resolved by our method, leading to most reads being rejected, we still see an increase in sensitivity at later stages of the run (Fig. 4a). This is due to BOSS-RUNS’ ability to sample more data specifically at positions where this is conducive to reducing uncertainty. For example, after 600 minutes of sequencing, BOSS-RUNS finds 26,481 variant sites in *P. aeruginosa* (sensitivity 0.79), whereas we observe 23,541 SNPs from control data (sensitivity 0.68), despite the decision strategy rejecting fragments from >80% of the genome after the first 180 minutes. At the same time, the precision of variant calls on BOSS-RUNS’ data is either moderately higher or at a similar level to the control (Fig. 4b). In the rarer species, the advantage of BOSS-RUNS simply collecting more data is evident, as we are able to call SNPs at least in some regions (119 and 80 SNPs after 600 minutes for *E. coli* and *S. enterica*, which make up 0.1% of total input DNA, respectively), whereas the control data do not contain enough reads to produce any variant calls.

Finally, we found no evidence that repeatedly rejecting molecules would have a negative impact on the performance of the section on the flowcell running BOSS-RUNS (Supplementary Figs. 4 and 5)

## Discussion

Our approach to dynamic, adaptive sampling for nanopore sequencing, implemented in BOSS-RUNS, provides a mathematical framework and fast algorithms to generate decision strategies that optimize the rate of information gain during resequencing experiments in real time. This leads to an increase in the sequencing yield of on-target regions, specifically at positions of highest uncertainty, and can effectively mitigate abundance bias or other sources of non-uniform coverage—for example, from enrichment library preparation procedures,— leading to smaller proportions of sites at low coverage depth and greater evenness of coverage. Furthermore, our methods lead to improved discovery of variants by both sampling more data from negatively biased regions or species in the input material and by focusing the sequencing on sites where the underlying genotype is not clear from the data observed up to that point in time.

Unlike existing adaptive sampling methods, our dynamic approach can change targets throughout an experiment to collect data where it is most useful. In common with any resequencing experiment, the only piece of prior knowledge that we require is a reference genome related to the organism(s) that we expect to observe in the sequenced material. Our method is, thus, potentially applicable to a wide array of biological problems, including studies of epigenetic modifications, which are now analyzable in real time with nanopore sequencing^22,23^. Additionally, it could harness the possibility of sequencing material other than genomic DNA, such as cDNA or RNA—for example, to correct abundance bias of transcripts. However, the shorter nature of fragments in these experiments and the presence of polyA tails at the start of sequenced fragments might reduce the potential benefit of BOSS-RUNS. Lastly, it could be used to overcome biases introduced by library preparation methods, such as exome pull-downs^24^.

The experiments presented have a mean read length of 3.11 kbp after amplification to achieve sufficiently high-molecular-weight DNA. This serves as a proxy for the challenging nature of extracting DNA from metagenomic samples, which often relies on harsh, multi-step procedures to ensure that cells from all contained species are lysed and genomic material is available for sequencing^25^. It was recently shown that average read length is a major determinant of the maximum level of enrichment using Read Until, with longer reads giving larger enrichment over the range of read lengths studied^26^. We would expect even greater benefits from dynamic adaptive sampling in experiments where longer reads were possible.

Depending on the underlying research question, a dynamic approach to adaptive sampling might not always be useful. For example, whereas our method inherently skews relative coverages in a mixture, accurate quantification remains possible by considering observed read counts instead (Supplementary Fig. 1). However, some experiments, such as detection of copy number variation, might require the preservation of underlying coverage information. Additionally, our current model does not account for complex variants, such as large insertions or deletions or low-frequency variants, and, thus, the potential benefit of sampling additional data at such sites might not be captured accurately. We, therefore, note that experiments with different aims might require different models within BOSS-RUNS, and we anticipate development of these in future extensions to our method.

Computational complexity of our algorithmic framework currently restricts the real-time application to prokaryotic or small eurakyotic genomes if every site of the genomes is modeled. Together with aforementioned anticipated improved models, generating strategies for entire genomes, while modeling and calculating positional expected benefit scores only for a priori known variant sites, might enable the use of our method during sequencing of much larger genomes.

In some scenarios, the need for reference genomes could also be a limitation. We are, therefore, working on extending our framework in a reference-free implementation that performs de novo assembly of the observed sequencing reads in real time. Dynamic strategies of such an approach could be used to fill gaps and extend the contiguity of existing assemblies or allow for true de novo enrichment of unknown genomes.

In conclusion, BOSS-RUNS expands the applicability of adaptive sampling and can improve the information gain in many standard scenarios. Using such data-driven strategies to ensure more homogeneous coverage and focusing on biologically interesting sites leads to improved efficiency of sequencing using nanopores. The resulting reduction in the time-to-answer or increased information gain might be critical in a clinical setting or in pathogen surveillance.

## Methods

### Probability distribution of genotypes at genomic sites

We define a probability distribution of possible genotypes at each position of one or multiple genomes. In brief, the genotype probability distribution takes both prior information about the genotype—for example, from a reference genome—and already observed bases at a position into account. Throughout, we use ‘reference genome’ to describe the assembly used for reference during a resequencing experiment and not necessarily the exact genome sequence of the investigated species.

Given already observed read data *D*, containing *n* reads covering position *i*, we denote by *d*_*j*,*i*_ ∈ *B*, with *B* = {A, C, G, T}, the nucleotide in read *j* that maps to *i*. For a haploid genome, the set of possible genotypes is *G* = *B*, whereas, for diploid genomes *G*, instead consists of unordered pairs *g* = {*b*_1_, *b*_2_}, with *b*_1_, *b*_2_ ∈ *B*. We define prior probabilities for genotype *g* at position *i* as *π*_*i*_(*g*), and the probability of calling base *d*_*j*,*i*_ assuming genotype *g* as *ϕ*(*d*_*j*,*i*_∣*g*), which represents a matrix of observation probabilities given assumptions about ploidy and sequencing errors (details in Supplementary Section 1.1). For simplicity, we present the case of genetic diversity and sequencing errors only occurring as SNPs—an extension that includes deletions and is used in our applications is provided in Supplementary Section 1.2. The posterior probability of genotype *g* ∈ *G* at *i*, conditional on *D*, is then

1 $${f}_{i}(g| D)=\frac{{\pi }_{i}(g)\mathop{\prod }\nolimits_{j = 1}^{n}\phi ({d}_{j,i}| g)}{{Z}_{i}(D)},$$

where *Z*_*i*_(*D*) represents a normalizing constant—that is, the likelihood of the data—that ensures the posterior probabilities sum to 1.

This model allows us to quantify the uncertainty about the genotype at each site (Fig. 1c) and, in turn, makes it possible to calculate the expected reduction in uncertainty resulting from observing a newly sequenced read. We call this expected reduction of uncertainty the ‘positional benefit score’ of a site. This quantity summarizes the expected change in the genotype probability distribution given one additional observation at that position and is calculated as follows: given the current data (*D*), we imagine that we observe one additional nucleotide *n* at position *i*—that is, *d*_*n*+1,*i*_—calling this augmented data *D*′. We then measure the difference between the distribution of genotype probabilities resulting from *D* and *D*′ by the Kullback–Leibler divergence (*D*_KL_; ref. ^27^).

Lastly, we sum over the different possible nucleotides *d*_*n*+1,*i*_, weighting their contributions by the estimated probability of observing them in the next read, to compute the expected reduction in uncertainty:

2 $${S}_{i}=\mathop{\sum}\limits_{{d}_{n+1,i}\in B}P({d}_{n+1,i}| D){D}_{{{{\rm{KL}}}}}({f}_{i}(g| D^{\prime} )\left\vert \right\vert {f}_{i}(g| D)),$$

where the estimated probability of observing nucleotide *d*_*n*+1,*i*_ in the next read is given by

3 $$P({d}_{n+1,i}| D)=\mathop{\sum}\limits_{g\in G}{f}_{i}(g| D)\phi ({d}_{n+1,i}| g).$$

A practical way of calculating the positional benefit scores and some examples at different coverage patterns are given in the Supplementary Material (Supplementary Section 1.3 and Supplementary Fig. 6). This technique of defining the information gain in terms of the Kullback–Leibler divergence of two distributions is used in Bayesian experimental design^28^ and is equivalent to evaluating the expected reduction in Shannon entropy^29^ brought by a new read.

### Estimating the expected benefit of sequencing reads

To quantify the potential information gain of future sequencing reads, we combine the positional benefit scores across sites that a sequencing read might span, to evaluate the expected benefit of such a read (Fig. 1d). We assume that a sequenced read will cover a number of consecutive sites of a reference genome equal to the molecule’s length *l*. The expected benefit is then calculated as the sum of consecutive positional scores, beginning from the read’s mapping starting position *i*, weighted by the distribution of previously observed read lengths, *L*(*l*). In other words, we form the sum ${S}_{i,o}^{l}$ of consecutive positional benefit scores of a read of length *l* starting at position *i* with orientation *o* (*o* = 1 indicating a read in the forward direction relative to the reference genome and 0 indicating the reverse direction); and then we combine these, weighted by the probability that the read will reach that position (Fig. 1d). For a forward-oriented read, ${S}_{i,1}^{l}$ will be

4 $${S}_{i,1}^{l}=\mathop{\sum }\limits_{j=i}^{i+l-1}{S}_{j}$$

(see Supplementary Section 1.4 for the reverse-oriented case), leading to the expected benefit

5 $${U}_{i,o}=\mathop{\sum}\limits_{l\in {{{{\mathcal{D}}}}}_{L}}L(l){S}_{i,o}^{l}.$$

Here, ${{{{\mathcal{D}}}}}_{L}$ represents the domain of *L*(*l*)—that is, all read lengths observed so far. In practice, we use a truncated normal distribution as a prior for read lengths, which we continuously update with observed lengths of full-length sequencing reads throughout an experiment. More details about *S*^*l*^ and *L*(*l*) are given in Supplementary Section 1.4.

With this, we can quantify the expected information gain of a sequencing read solely on the basis of its genomic origin and orientation. We provide an approximation to calculate this quantity based on a piece-wise approximation of the read length distribution in Supplementary Section 1.4.

### Optimal strategies to maximize rate of information gain

To define our decision strategies, we parameterize the duration of individual steps in the sequencing process. As our time unit, we use the amount of time it takes one base to translocate through a pore (Fig. 1f). Analogous to Read Until and readfish, we start sequencing a DNA fragment and use *μ* initial bases to determine its genomic origin and orientation. The value of *μ* is assumed constant in our model and can be adjusted to ensure mappings of sufficient quality—for example, depending on the complexity or repeat content of the used reference genome. In practice, *μ* depends on the size of individual data chunks used for real-time basecalling. The smallest useful setting is 0.4 seconds of input data, which corresponds to ~180 nt, assuming a translocation speed of 450 nt s^−1^. In our applications, we used 0.8 seconds of data and observed a mean length of 348 nt for real-time basecalled data chunks used to determine the origin of fragments. We further assume that some constant time is needed to effect the rejection of a read (*ρ*) and to acquire a new read at a pore (*α*). In line with measurements from sequencing experiments, our model assumes *ρ* = 300 and *α* = 300 by default. If a fragment is sequenced fully, time equal to its length *l* passes, and benefit ${S}_{i,o}^{l}$ is accrued (with expectations *λ* = E[*L*] and *U*_*i*,*o*_, respectively); by rejecting a read, time equal to *l* − *μ* − *ρ* can be saved, and the expected gain of benefit is limited to the positional scores of its initial fragment—that is, ${S}_{i,o}^{\mu }$ (Fig. 1f and Supplementary Fig. 7).

With this parameterization of the sequencing process, we determine an optimal sequencing strategy that maximizes the expected benefit per unit of sequencing time given the currently available data. Such a strategy, denoted as ${\mathcal{S}}$, can be seen as an indicator function that returns 0 (reject) or 1 (accept) for all combinations of genomic position and fragment orientation—for example, ${I}_{i,1}^{{{{\mathcal{S}}}}}=0$ indicates the rejection of a forward-oriented read at position *i*, and ${I}_{i,0}^{{{{\mathcal{S}}}}}=1$ is the acceptance of a reverse-oriented read. Our aim is, therefore, to find an optimal strategy $\widehat{\mathcal{S}}$ that maximizes the benefit per unit time ${\overline{U}}^{{{{\mathcal{S}}}}}/{\bar{t}}^{{{{\mathcal{S}}}}}$ given the current data *D*:

6 $$\widehat{{{{\mathcal{S}}}}}=\arg \mathop{\max }\limits_{{{{\mathcal{S}}}}}\frac{{\overline{U}}^{{{{\mathcal{S}}}}}}{{\bar{t}}^{{{{\mathcal{S}}}}}}.$$

Here, ${\overline{U}}^{{{{\mathcal{S}}}}}$ is the average expected benefit. Given a genome with a total length *N* and the average expected benefit of the initial parts of reads—that is, the benefit ${\bar{S}}^{\mu }$ accrued from the initial fragment used in the decision process—it takes the form

7 $${\overline{U}}^{{{{\mathcal{S}}}}}={\bar{S}}^{\mu }+\frac{1}{2N}\mathop{\sum}\limits_{o=1,0}\hspace{2.22144pt}\mathop{\sum }\limits_{i=1}^{N}{I}_{i,o}^{{{{\mathcal{S}}}}}\left({U}_{i,o}-{S}_{i,o}^{\mu }\right).$$

In other words, it is the sum of the average expected benefit from a read of *μ* bases and the average of a fully sequenced read, which adds further benefit of ${U}_{i,o}-{S}_{i,o}^{\mu }$ if the indicator function for that position–orientation combination returns 1. Then, ${\bar{t}}^{{{{\mathcal{S}}}}}$ is the expected time needed to complete the processing (whether accepted or rejected) of a read:

8 $${\bar{t}}^{{{{\mathcal{S}}}}}=\alpha +\mu +\rho +\frac{| {{{\mathcal{S}}}}| }{2N}(\lambda -\mu -\rho )\hspace{2.22144pt},$$

where $| {{{\mathcal{S}}}}|$ denotes the size of the strategy—that is the number of position–orientation pairs for which the indicator function will return 1—and *λ* is the mean read length (E[*L*], as above).

For simplicity, here we assume uniformity of the distribution of read origins; we present a generalization used in our implementation in Supplementary Sections 1.5 and 1.6.

To compute the optimal strategy, we rank all of the position–orientation combinations (*i*, *o*) in decreasing order of ${U}_{i,o}-{S}_{i,o}^{\mu }$, the expected benefit gain from sequencing them in their entirety. Starting with an empty strategy (one that rejects all reads), we successively include the ranked sites and test after each one whether its contribution results in an improvement over the previous strategy—that is, whether the current iteration achieves higher gain of benefit per time unit (${\overline{U}}^{{{{\mathcal{S}}}}}/{\bar{t}}^{{{{\mathcal{S}}}}}$) than the preceding strategy that included one fewer site (position–orientation pair). For an overview of parameters and variables in the model and proof of optimality, see Supplementary Sections 1.5 and 1.7 and Supplementary Table 1.

### Implementation details

Effecting decisions about reads is performed by a modified version of readfish^14^, which uses our dynamically updated strategies throughout an experiment. It is available at https://github.com/LooseLab/readfish/tree/BossRuns/V0.0.2.

For taking newly observed reads into account, we consider only one possible mapping to the reference(s). Therefore, if a read maps to more than one position, the best alignment is chosen based on mapping quality or the alignment score of the dynamic programming algorithm in case of a tie. Observed lengths of fully sequenced reads and their mapping positions are continuously used to update the empirical distributions of read lengths *L*(*l*) and read start locations and orientations (Supplementary Section 1.6). To prevent the strategy from getting too greedy, updates are applied only when a region surpasses a threshold of average coverage (default: ≥5× in 20-kb windows). To keep pace with the real-time data stream and to ensure optimality of the strategy at any point in time, new results need to be calculated quickly. We use several optimizations, including an algorithm to find approximate decision strategies, which are described in Supplementary Section 1.8.

Our method can use either single or multiple reference chromosomes/genomes as input and optional masks to indicate initial ROIs, similarly to current approaches to adaptive sampling^14,26^. In that case, the scope of the dynamically updated strategies is limited to the ROIs and flanking regions around them; reads originating outside these regions will always be rejected. If multiple references are considered, the expected benefit of reads is calculated separately per reference and then used to derive a common decision strategy across all considered references. This ensures that we can account for differences in the distributions of read lengths and read starting positions between genomes while also sequencing the most informative reads of a mixture, instead of focusing on the most informative reads of each individual genome or chromosome.

BOSS-RUNS is implemented in Python and available at https://github.com/goldman-gp-ebi/BOSS-RUNS. We provide a conda environment for its dependencies (with most recent tested versions denoted): readfish^14^, ONT’s MinKnow API 5.0.0.1 (ref. ^30^), numpy 1.22.4 (ref. ^31^), numba 0.55.2 (ref. ^32^), scipy 1.9.0 (ref. ^33^), mappy 2.24 (ref. ^19^), pandas 1.4.3 (ref. ^34^), toml 0.10.2 (ref. ^35^) and natsort 8.1.0 (ref. ^36^).

### Configuration of sequencing experiments

Sequencing was conducted on an ONT GridION using R9.4 flowcells. Because the quality and number of active nanopores can vary between flowcells, it would be difficult to compare experiments involving adaptive sampling performed on multiple flowcells. Therefore, we separated a single flowcell by assigning 256 channels to each of two different conditions. One of these two regions used a decision strategy that continuously accepts any encountered read—that is, a control sector not performing any adaptive sampling—whereas the other was acting according to the decision strategies provided by BOSS-RUNS. A heat map of the yield per channel as well as spatial autocorrelation statistics confirm that the loading and splitting of the flowcell did not influence the results of our experiment (Supplementary Fig. 8).

Readfish was configured to reject reads from the sector analyzed using BOSS-RUNS if they did not map or mapped to (one or more) off-target sites—that is, sites not included in the current decision strategy—or if no sequence was obtained from a fragment. For all our experiments, we used 0.8 seconds of data to infer the genomic origin and orientation of fragments before making decisions—that is, roughly 350 bp (corresponding to *μ* in our model; Fig. 1f), which results in a mean read length of 482 bp for rejected reads due also to the additional time (*ρ*) taken to process and effect decisions. BOSS-RUNS deposits new strategies as compressed Boolean numpy arrays for each genome or chromosome, which are subsequently reloaded by readfish upon file modification. Communicating rejection signals to the sequencing device is performed by readfish.

### Sequencing of the ZymoBIOMICS microbial reference

Input DNA from the ZymoBIOMICS Microbial Community DNA Standard II (Log Distribution D6311, Zymo Research) was prepared using SQK-LSK110 (ONT) and PCR-amplified using the PCR expansion kit EXP-PCA001 (ONT). BOSS-RUNS and readfish depend on reference genomes to infer the origin of sequencing reads. To mimic a more realistic scenario where we do not know the exact bacterial strains, we elected not to use reference genomes from the strains contained in the microbial mixture but, instead, used closely related reference genomes identified in ref. ^37^. We measured their divergence in terms of the percentage of aligning nucleotides and ANI values using JSpecies^38^, which range from 86.07% to 99.70% and 98.82% to 99.92%, respectively (Supplementary Fig. 9). The employed assemblies are available in the European Nucleotide Archive (ENA) under accession numbers ASM14656v1, ASM584v2, ASM400627v1, ASM39716v1, ASM30761v1, ASM51030v1, ASM25313v1 and ASM810v1. Software used during data collection included MinKNOW (21.05.25), MinKNOW core (4.3.12), MinKNOW api (5.0.0.1) and Bream (6.2.6). Basecalling was performed using Guppy (5.0.16), set to high-accuracy mode. The sequencing data generated in this study are available in the ENA database under accession number PRJEB51967.

To test whether increased coverage of rare species was due to repeats or low-complexity regions, we used RepeatMasker 4.1.2 with default parameters^39^.

### Variant calling of bacterial species

To perform variant calling, we used sequencing reads separated by their species of origin (using minimap2 (ref. ^19^)) and further partitioned them to comprise the cumulative data from the beginning of the experiment up to and including 20 individual timepoints, each separated by approximately 30 minutes of sequencing (using custom Python scripts).

To create a set of high-confidence variants, we used publicly available deep coverage short-read sequencing of the ZymoBIOMICS microbial community with evenly distributed abundances, which contains the same strains as the logarithmically distributed mixture (Zymo Research, D6306). These data are available in the ENA under accession number SRR13224035. In brief, we mapped the separated reads to their respective assemblies (see previous section) using minimap2 2.22 (ref. ^19^) and samtools 1.12 (ref. ^40^), marked duplicates using picard 2.26.6 (default parameters)^41^ and called variants—that is, the differences between the assemblies that we used and the strains contained in the sequenced microbial community—with freebayes 1.3.5 (default parameters)^42^. Variants were filtered by minimum depth of coverage of 20 and quality score of 20, transformed into their primitive constituents (vcflib 1.0.2 (ref. ^43^)) and sorted using bcftools 1.12 (ref. ^40^). Variant calling from nanopore data of the Zymo microbial mixture was done using medaka 1.4.3 (default parameters, model r941_prom_hac_variant_g507)^44^. For subsequent comparisons of vcf files, we used vcfeval (rtg-tools 3.12.1 (ref. ^45^)).

### Reporting summary

Further information on research design is available in the Nature Portfolio Reporting Summary linked to this article.

## Online content

Any methods, additional references, Nature Portfolio reporting summaries, source data, extended data, supplementary information, acknowledgements, peer review information; details of author contributions and competing interests; and statements of data and code availability are available at 10.1038/s41587-022-01580-z.

## Supplementary information

Supplementary Information Supplementary Methods (Sections 1.1–1.8), Supplementary Results (Section 2.1), Supplementary Figs. 1–12 (Section 3), Supplementary Tables 1 and 2 (Section 4) and References

Reporting Summary

## Data Availability

The sequencing data generated in this study have been submitted to the ENA database under accession number PRJEB51967. Publicly available assemblies and short-read sequencing data used in our study are available in the ENA under accession numbers ASM14656v1, ASM584v2, ASM400627v1, ASM39716v1, ASM30761v1, ASM51030v1, ASM25313v1 and ASM810v1 as well as SRR13224035.

## Code availability

The source code of BOSS-RUNS is available at https://github.com/goldman-gp-ebi/BOSS-RUNS.
