# Supplementary material for: Dynamic, adaptive sampling during nanopore sequencing using Bayesian experimental design
Source: Nat Biotechnol. 2023 Jan 2;41(7):1018–25. doi: 10.1038/s41587-022-01580-z (PMC10344778; doi:10.1038/s41587-022-01580-z)
Supplement: Supplementary file 2 — Reporting Summary [file 41587_2022_1580_MOESM2_ESM.pdf]

nature portfolio

Corresponding author(s): Nick Goldman

Last updated by author(s): Oct 12, 2022

Reporting Summary

Nature Portfolio wishes to improve the reproducibility of the work that we publish. This form provides structure for consistency and transparency in reporting. For further information on Nature Portfolio policies, see our Editorial Policies and the Editorial Policy Checklist.

Statistics

For all statistical analyses, confirm that the following items are present in the figure legend, table legend, main text, or Methods section.

n/a	Confirmed	
☐	☒	The exact sample size (*n*) for each experimental group/condition, given as a discrete number and unit of measurement
☐	☒	A statement on whether measurements were taken from distinct samples or whether the same sample was measured repeatedly
☐	☒	The statistical test(s) used AND whether they are one- or two-sided<br>*Only common tests should be described solely by name; describe more complex techniques in the Methods section.*
☐	☒	A description of all covariates tested
☐	☒	A description of any assumptions or corrections, such as tests of normality and adjustment for multiple comparisons
☐	☒	A full description of the statistical parameters including central tendency (e.g. means) or other basic estimates (e.g. regression coefficient) AND variation (e.g. standard deviation) or associated estimates of uncertainty (e.g. confidence intervals)
☐	☒	For null hypothesis testing, the test statistic (e.g. F, t, r) with confidence intervals, effect sizes, degrees of freedom and P value noted<br>*Give P values as exact values whenever suitable.*
☐	☒	For Bayesian analysis, information on the choice of priors and Markov chain Monte Carlo settings
☒	☐	For hierarchical and complex designs, identification of the appropriate level for tests and full reporting of outcomes
☒	☐	Estimates of effect sizes (e.g. Cohen's *d*, Pearson's *r*), indicating how they were calculated

Our web collection on statistics for biologists contains articles on many of the points above.

Software and code

Policy information about availability of computer code

Data collection

All software used for data collection is fully referenced in the ms. This comprises:

ONT's software:
MinKNOW 21.05.25
MinKNOW core 4.3.12
MinKNOW api 5.0.0.1
Bream 6.2.6
Guppy 5.0.16

Open source software:
readfish (commit e86c65fe57a9691fcb6a9a7755545b25f9fc38d9)
https://github.com/LooseLab/readfish/tree/e86c65fe57a9691fcb6a9a7755545b25f9fc38d9

Software devised and described in this study:
BOSS-RUNS (commit f58c4ddb8446530857ada226ece366d89f9a05b3)
https://github.com/goldman-gp-ebi/BOSS-RUNS/tree/f58c4ddb8446530857ada226ece366d89f9a05b3

Data analysis

All software used for data analysis is fully referenced in the ms., either as a dependency of BOSS-RUNS (see above) or in the methods section.

This comprises the following (all open source):

numpy 1.22.4
numba 0.55.2
scipy 1.9.0
mappy 2.24
minimap2 2.22
pandas 1.4.3
toml 0.10.2
natsort 8.1.0
rtg-tools 3.12.1
samtools 1.12
bcftools 1.12
picard 2.26.6
freebayes 1.3.5
medaka 1.4.3
vcflib 1.0.2
JSpeciesWS 3.9.3
RepeatMasker 4.1.2

Additional widely known open source software used for data analysis and visualization:

matplotlib 3.4.2
plotnine 0.8.0
python-ternary 1.0.8
pysam 0.16.0.1
python 3.9.6
r-base 4.1.0
ggplot2 3.3.5
plyr 1.8.6
ggridges 0.5.2
snakemake 6.6.0
PySAL 2.6.0
GeoPandas 0.11.0

For manuscripts utilizing custom algorithms or software that are central to the research but not yet described in published literature, software must be made available to editors and reviewers. We strongly encourage code deposition in a community repository (e.g. GitHub). See the Nature Portfolio guidelines for submitting code & software for further information.

Data

Policy information about availability of data

All manuscripts must include a data availability statement. This statement should provide the following information, where applicable:

- Accession codes, unique identifiers, or web links for publicly available datasets
- A description of any restrictions on data availability
- For clinical datasets or third party data, please ensure that the statement adheres to our policy

All the data we have used is publicly available, including both pre-existing data generated by others and novel data generated in the course of our study. All datasets used have associated statements of availability, for example pre-existing microbial reference assemblies ("The employed assemblies are available in the European Nucleotide Archive under accessions ASM14656v1, ASM584v2, ASM400627v1, ASM39716v1, ASM30761v1, ASM51030v1, ASM25313v1, ASM810v1."); pre-existing short-read sequencing data from analysis of the ZymoBIOMICS microbial community ("These data are available in the European Nucleotide Archive under the accession SRR13224035."); and nanopore sequencing read data generated by us ("The sequencing data generated in this study have been submitted to the ENA database under accession number PRJEB51967.").

Human research participants

Policy information about studies involving human research participants and Sex and Gender in Research.

Reporting on sex and gender	No human research participants. Paper concerns only microbial species and so no sex or gender consideration is relevant or possible.
Population characteristics	No human research participants.
Recruitment	No human research participants.
Ethics oversight	No oversight necessary as there were no human research participants.

Note that full information on the approval of the study protocol must also be provided in the manuscript.

Field-specific reporting

Please select the one below that is the best fit for your research. If you are not sure, read the appropriate sections before making your selection.

☒ Life sciences ☐ Behavioural & social sciences ☐ Ecological, evolutionary & environmental sciences

For a reference copy of the document with all sections, see nature.com/documents/nr-reporting-summary-flat.pdf

Life sciences study design

All studies must disclose on these points even when the disclosure is negative.

Sample size	Sequencing of a microbial mixture (ZymoBIOMICS DNA Standard II D6311, Zymo Research) was carried out as described in the ms. One sample of the microbial mixture was used for sequencing. No further samples are needed to prove efficacy of methods forming the results of our paper.
Data exclusions	No data were excluded.
Replication	Implicit replication was provided by the parallel nature of the many flowcell nanopores used for each of the test and control conditions. Each nanopore was verified to behave as expected given our dynamically updated selection strategies by checking the most likely source organism of each observed read and the expected decision about that fragment. All findings were thus replicated over all the pores.
Randomization	The microbial mixture sequenced is a representative reference sample to test methodologies. The outcome of the experiment is the success or failure of the method. Since we sequenced one sample no allocation into experimental groups was performed.
Blinding	The sample is a commercially available reference and is not amenable to blinding. It was selected specifically because its content is well-known, allowing for validation of our method.

Reporting for specific materials, systems and methods

We require information from authors about some types of materials, experimental systems and methods used in many studies. Here, indicate whether each material, system or method listed is relevant to your study. If you are not sure if a list item applies to your research, read the appropriate section before selecting a response.

Materials & experimental systems

n/a	Involved in the study	
☒	☐	Antibodies
☒	☐	Eukaryotic cell lines
☒	☐	Palaeontology and archaeology
☒	☐	Animals and other organisms
☒	☐	Clinical data
☒	☐	Dual use research of concern

Methods

n/a	Involved in the study	
☒	☐	ChIP-seq
☒	☐	Flow cytometry
☒	☐	MRI-based neuroimaging
